# The supercontinent cycle and Earth's long‐term climate

**DOI:** 10.1111/nyas.14849

**Published:** 2022-06-28

**Authors:** R. Damian Nance

**Affiliations:** ^1^ Department of Geological Sciences Ohio University Athens Ohio USA; ^2^ Department of Earth & Planetary Sciences Yale University New Haven Connecticut USA; ^3^ Institute of Geology and Palaeontology Charles University Prague 2 Czech Republic

**Keywords:** atmospheric CO_2_, climate, large igneous provinces, sea level, supercontinent cycle

## Abstract

Earth's long‐term climate has been profoundly influenced by the episodic assembly and breakup of supercontinents at intervals of ca. 500 m.y. This reflects the cycle's impact on global sea level and atmospheric CO_2_ (and other greenhouse gases), the levels of which have fluctuated in response to variations in input from volcanism and removal (as carbonate) by the chemical weathering of silicate minerals. Supercontinent amalgamation tends to coincide with climatic cooling due to drawdown of atmospheric CO_2_ through enhanced weathering of the orogens of supercontinent assembly and a thermally uplifted supercontinent. Conversely, breakup tends to coincide with increased atmospheric CO_2_ and global warming as the dispersing continental fragments cool and subside, and weathering decreases as sea level rises. Supercontinents may also influence global climate through their causal connection to mantle plumes and large igneous provinces (LIPs) linked to their breakup. LIPs may amplify the warming trend of breakup by releasing greenhouse gases or may cause cooling and glaciation through sulfate aerosol release and drawdown of CO_2_ through the chemical weathering of LIP basalts. Hence, Earth's long‐term climatic trends likely reflect the cycle's influence on sea level, as evidenced by Pangea, whereas its influence on LIP volcanism may have orchestrated between Earth's various climatic states.

## Graphical abstract

The supercontinent cycle, by which Earth history is viewed as having been punctuated by the episodic assembly and breakup of supercontinents, has, through its management of plate motion, planetary geography, sea level and mantle circulation, profoundly influenced Earth's long‐term climatic history. It is likely that the supercontinent cycle has, over the course of Earth history, played a dominant role in governing the climate of individual continents, the planet's long‐term warming and cooling trends, and its occasional climatic extremes, while, at the same time, maintaining surface conditions sufficiently hospitable to ensure the continuity of life.

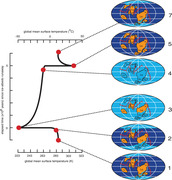

## INTRODUCTION

**FIGURE 1:**
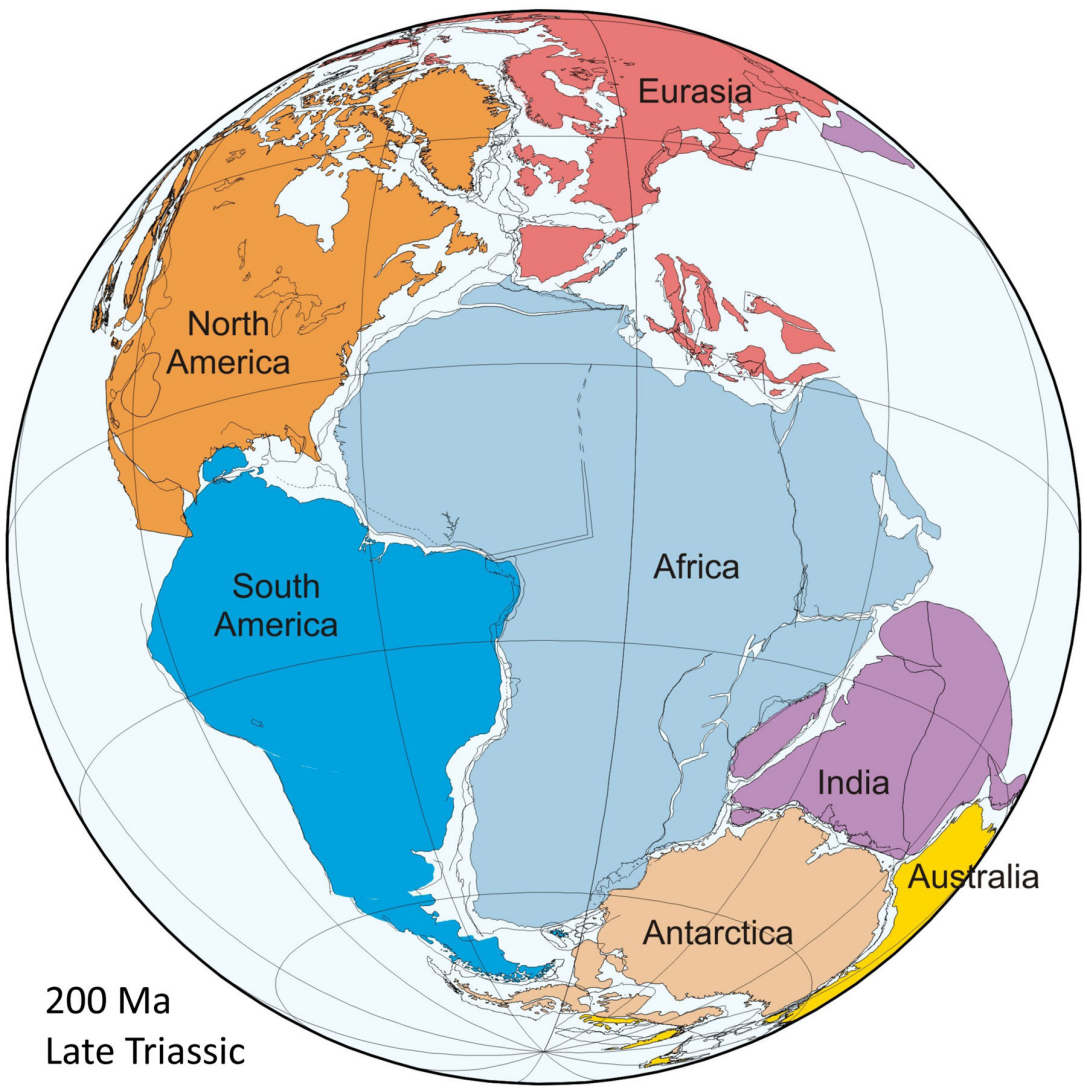
Reconstruction of Pangea for the Late Triassic (at 200 Ma) by the PLATES program at the University of Texas Institute of Geophysics. (http://www‐udc.ig.utexas.edu/external/plates/images/pangea_07sep2007.jpg)

**FIGURE 2:**
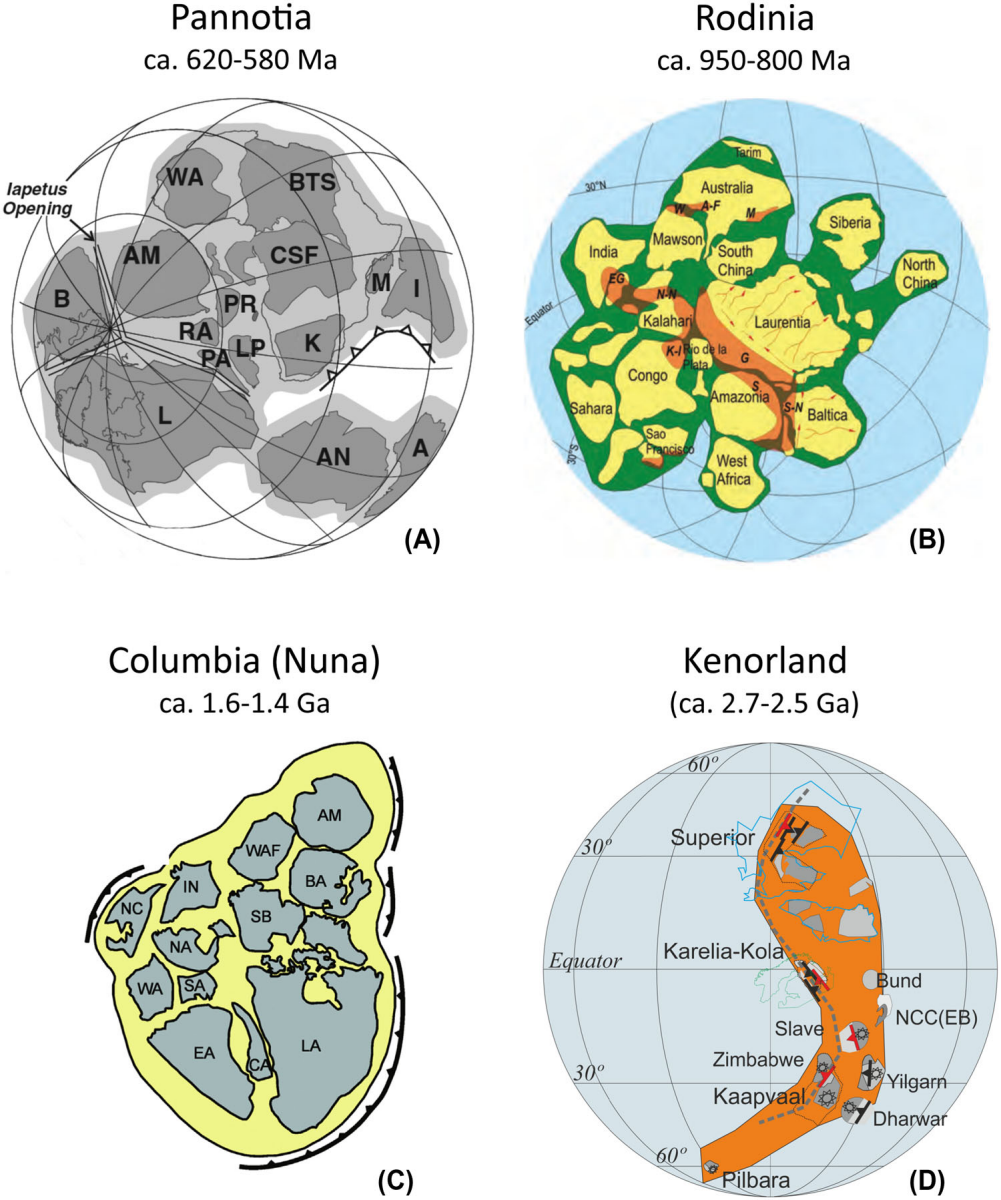
Proposed reconstructions of pre‐Pangean supercontinents. (A) Pannotia (c. 620–580 Ma^238^), (B) Rodinia (c. 950–800 Ma^239^ simplified after Li *et al*.^17^), (C) Nuna/Columbia (c. 1.6–1.4 Ga^23^), and (D) Kenorland (c. 2.7–2.5 Ga). Abbreviations: (A) A, Australia; AM, Amazonia; AN, Antarctica; B, Baltica; BTS, Borborema–Trans‐Sahara; CSF, Congo–São Francisco; I, India; K, Kalahari; L, Laurentia; LP, Rio de la Plata; M, Madagascar; PA, Pampea; PR, Paraná; RA, Rio Apa; WA, West Africa. (B) A‐F, Albany‐Fraser orogen; EG, Eastern Ghats belt; K‐I, Kibaran and Irumide belts; M, Musgrave orogen; N‐N, Namaqua‐Natal province; S, Sunsas orogen; S‐N, Sveco‐Norwegian orogen; W, Wilkes province. (C) AM, Amazonia; BA, Baltica; CA, Cathaysia; EA, East Antarctica; LA, Laurentia; IN, India; NC, North China; NA, North Australia; SA, South Australia; SB, Siberia; WA, West Australia; WAF, West Africa. (D) Bund, Bundelkhand craton; NCC(EB), Eastern block of North China craton

The supercontinent cycle describes the realization, developed over the past 30 years, that much of Earth history has been punctuated by the episodic assembly and breakup of supercontinents, during which most of Earth's continents are assembled into a single landmass.^1^ Consequently, the well‐documented supercontinent Pangea (Figure 1), first advocated by Wegener,^2^, ^3^ is viewed as only the most recent in a series of supercontinents that have assembled and broken up at intervals of roughly half‐a‐billion years since perhaps as far back as the late Archean.^4^, ^5^, ^6^, ^7^, ^8^ Major support for this hypothesis has come with the recognition of supercontinents (Figure 2) at c. 620–580 Ma (*Pannotia*,^6^, ^9^, ^10^, ^11^, ^12^ the existent of which is debated^13^, ^14^, ^15^), c. 950–800 Ma (*Rodinia* ^6^, ^16^, ^17^, ^18^), and c. 1.6–1.4 Ga (*Nuna* or *Columbia* ^19^, ^20^, ^21^, ^22^, ^23^, ^24^), and possible supercontinents (or supercratons) at c. 2.7–2.5 Ga (*Kenorland*;^25^, ^26^, ^27^ *Lauroscandia* ^28^) and c. 3.0 Ga (*Ur* ^29^, ^30^), in addition to Wegener's *Pangea* (c. 325–200 Ma).

The episodic cycle has been linked to global orogenesis,^31^, ^32^, ^33^ granitoid magmatism and zircon age peaks,^34^, ^35^, ^36^, ^37^ crustal growth,^38^, ^39^, ^40^, ^41^ mineralization,^42^, ^43^, ^44^, ^45^, ^46^, ^47^, ^48^ large igneous provinces (LIPs)^49^, ^50^, ^51^, ^52^, ^53^ and deep mantle convection patterns.^54^, ^55^, ^56^, ^57^, ^58^, ^59^, ^60^ Additionally, the cycle has been shown to have profound affects on sea level,^61^, ^62^, ^63^, ^64^, ^65^, ^66^ ocean chemistry,^35^, ^67^, ^68^, ^69^ the stable isotope record,^35^, ^70^, ^71^, ^72^ patterns of sedimentation,^73^, ^74^, ^75^ atmospheric composition,^76^, ^77^, ^78^ global biogeochemical cycles,^4^, ^79^, ^80^ climate^74^, ^81^, ^82^, ^83^, ^84^ marine biodiversity,^85^, ^86^ and the evolution of life.^83^, ^87^, ^88^

The supercontinent cycle is consequently a unifying hypothesis with major implications for the geosciences and our understanding of Earth's evolution. It has likely influenced the rock record more than any other geologic phenomena,^89^ its existence documents fundamental processes in the Earth's mantle and at the core–mantle boundary,^50^ and it has probably governed the planet's surface environment for much of Earth history.^90^

For detailed reviews of the history, development, and consequences of the supercontinent cycle, the reader is referred to Nance and Murphy^91^ and Nance *et al*.^1^ Here, I focus on just one aspect of the cycle—its affect on Earth's climate and climate‐controlling processes.

## BACKGROUND

That Earth history may have been punctuated by the episodic assembly and breakup of supercontinents with profound consequences to the geosphere is not a new idea,^4^, ^5^, ^61^, ^79^, ^92^ and the notion of long‐term episodicity in tectonic processes predates plate tectonics.^93^, ^94^, ^95^, ^96^, ^97^, ^98^, ^99^, ^100^ However, widespread recognition of the supercontinent cycle is a relatively recent phenomenon,^7^, ^8^, ^55^, ^89^, ^101^ as is the growing consensus regarding its profound effect on Earth history and evolution.^40^, ^80^, ^81^, ^83^, ^90^, ^102^, ^103^, ^104^, ^105^

**FIGURE 3:**
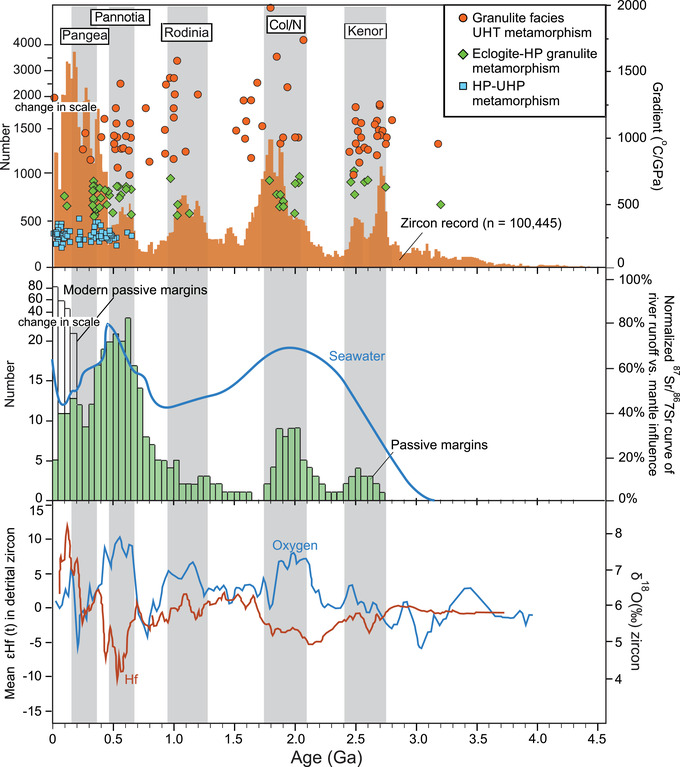
Secular trends in detrital zircon ages, granulite facies thermal gradients, passive margin development, normalized seawater ^87^Sr/^86^Sr, and mean initial εHf and average δ^18^O in detrital zircons from recent sediments compared with the assembly (shaded intervals) of various proposed pre‐Pangean supercontinents (from Hawkesworth *et al*.^36^ and references therein). Abbreviations: HP, high pressure; UHP, ultra‐high pressure; UHT, ultra‐high temperature

A wide variety of phenomena have been linked to the supercontinent cycle (Figure 3). Supercontinent assembly, for example, is accompanied by terrane accretion, collisional orogenesis, and continental shortening as the continents amalgamate and the oceans between them close.^39^ Orogenic granitoid magmatism, recorded as U–Pb age peaks for zircons with evolved εHf and elevated δ^18^O values consistent with increased reworking of crustal and sedimentary material, is enhanced,^34^, ^35^, ^41^, ^106^, ^107^ as are conditions for continental arc magmatism,^108^ extreme (UHT and UHP) metamorphism,^32^, ^109^ and active margin sedimentation with high clastic to carbonate ratios.^75^ Epeirogenic uplift through continental insulation and mantle upwelling, both of which are thought to be consequences of supercontinent amalgamation,^56^, ^110^, ^111^ lead to a global lowering of sea level^63^, ^64^, ^112^, ^113^ with accompanying enhanced weathering and terrestrial deposition.^76^ The resulting drawdown of atmospheric CO_2_ causes climatic cooling,^114^, ^115^ while the loss of insular continents and shallow‐marine habitats leads to low biotic diversity^85^ and may precipitate mass extinctions. Enhanced erosion increases seawater ^87^Sr/^86^Sr, δ^34^S and nutrient supply,^35^, ^70^, ^71^, ^72^, ^116^, ^117^ while the resulting rise in marine productivity and photosynthesis acts to increase atmospheric oxygen levels.^71^, ^78^

On the other hand, supercontinent breakup and dispersal reverses many of these trends and is heralded by peripheral subduction rollback^50^, ^118^, ^119^, ^120^ and continental rifting documented in mafic dike swarms and LIPs,^49^, ^51^, ^53^, ^121^ followed by passive margin development.^75^, ^122^ Subdued collisional orogeny and granitoid magmatism is recorded in troughs in U–Pb age spectra for zircons with juvenile εHf and lowered δ^18^O values consistent with increased mantle‐derived magmatism.^34^, ^35^, ^41^, ^107^ Thermal subsidence and extension of the dispersing continental fragments, and the creation of a younger world ocean floor through the opening of new ocean basins and consequent increase in ridge length, is accompanied by rapid sea level rise,^63^, ^64^, ^66^, ^123^ enhanced shallow marine sedimentation,^90^ and organic carbon burial leading to negative δ^13^C anomalies.^70^, ^83^ Diminished seawater ^87^Sr/^86^Sr ratios and warm, equable climates are linked to elevated atmospheric CO_2_ levels, driving rapid evolutionary radiation of new taxa and increasing biotic diversity.^35^, ^70^, ^71^, ^78^, ^124^

The cycle is likely driven by some combination of continental insulation, mantle plume dynamics, and slab rollback. The mechanism first proposed was that of continental insulation,^61^, ^110^, ^125^ whereby the thermal insulating effect of continental lithosphere on mantle heat flow is considered to trap mantle heat beneath supercontinents resulting in their thermal uplift and breakup.^56^, ^111^, ^126^ The new oceans so produced then either widen until the leading edges of the dispersing continental fragments collide to form a new supercontinent, a process termed extroversion,^127^ or they close as their floors grow older and less buoyant, such that the continental fragments are reassembled, a process termed introversion. In both cases, the assembled supercontinent would once again trap mantle heat and the cycle would be repeated.

**FIGURE 4:**
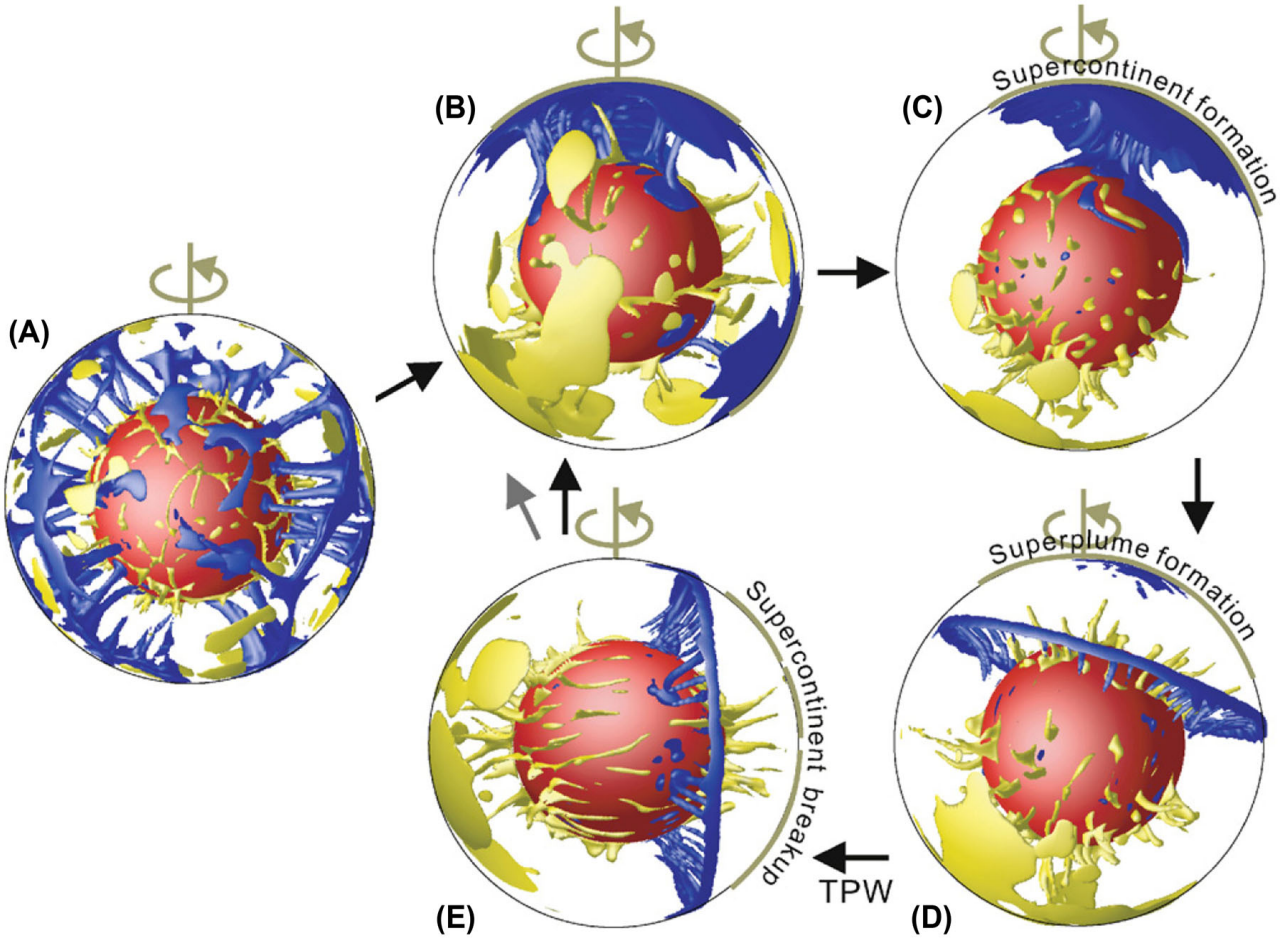
Numerical modeling of supercontinent assembly and breakup.^50^ (A) Initial small‐scale convection evolves to (B) an early stage degree‐1 mantle structure (antipodal regions of upwelling and downwelling) as the supercontinent assembles, and (C) a stable degree‐1 structure as the supercontinent forms. (D) With the formation of a subduction girdle and the onset of a superplume beneath the supercontinent, convection evolves to a degree‐2 planform (antipodal regions of upwelling), which (E) contributes to supercontinent breakup as true polar wander brings the supercontinent to the equator. Alternation of the two modes of mantle convection is thought to be responsible for the cyclic process of supercontinent assembly and breakup. Blue = cool mantle, yellow = hot mantle, red = core

Alternatively, the mechanism may be a consequence of the cycle's strong coupling to mantle dynamics,^60^ whereby subduction to the core–mantle boundary of the oceanic lithosphere surrounding a supercontinent creates mantle plumes that rise beneath them and contribute to their breakup.^38^, ^50^, ^54^, ^128^ In this case (Figure 4), supercontinents are considered to form over areas of mantle downwelling in an Earth with a degree‐1 mantle structure, that is, one with single, antipodal zones of mantle upwelling and downwelling.^54^ They subsequently break up because the subduction girdle that develops around a supercontinent once it assembles creates a slab graveyard of subducted oceanic lithosphere at the core–mantle boundary,^129^, ^130^ which influences the mantle's large low shear velocity provinces (LLSVPs) in such a way as to foster the generation of mantle plumes that rise beneath the supercontinent.^50^, ^59^, ^60^, ^131^ The result is an Earth with a degree‐2 mantle structure, that is, one with two antipodal zones of upwelling, the one beneath the supercontinent being responsible for its breakup.^50^, ^54^ Upon breakup, the subduction girdle that develops around the supercontinent following its assembly forms a new ring of mantle downwelling over which the dispersing continental fragments gather. This girdle, which would be longitudinal if true polar wander brings a supercontinent to the equator,^50^, ^54^, ^132^ may then move away from the former supercontinent to recreate an antipodal degree‐1 mantle structure and reassemble a supercontinent by way of extroversion, or it may move toward the former supercontinent and reassemble one by way of introversion.^127^ Alternatively, the dispersing continental fragments may coalesce along the girdle such that the new supercontinent assembles roughly 90 degrees away from its predecessor, a process termed orthoversion.^133^

A potential breakup mechanism also exists in the forces associated with slab rollback along the supercontinent periphery.^118^, ^120^, ^134^, ^135^, ^136^ This mechanism is consistent with the development of a slab girdle, the oceanward retreat of which would generate extensional forces that may be sufficient to cause supercontinent breakup.^118^

All three mechanisms are supported by modeling,^55^, ^118^, ^125^, ^126^ and it is likely that each plays a role in the breakup of supercontinents once they have amalgamated. Hence, the cycle appears to operate because supercontinents sow the seeds of their own destruction and break up, but in doing so, they set the stage for their eventual reassembly. While their relationship to the supercontinent cycle is unlikely to be a simple one,^52^, ^137^, ^138^ the apparent role of mantle plumes is significant because it links the supercontinent cycle to deep mantle upwelling and processes occurring at the core–mantle boundary. Hence, it elevates the supercontinent cycle from a near‐surface phenomena to a whole‐mantle process linking top‐down plate tectonics and bottom‐up plume tectonics.

## INFLUENCE ON GLOBAL CLIMATE

The role of the supercontinent cycle in governing long‐term global climate is chiefly based on the Phanerozoic record and rests largely on its influence on global sea level and the governing affect this has on continental erosion and silicate weathering, and the consequent abundance of CO_2_ and other greenhouse gases in the atmosphere.^4^, ^76^, ^139^, ^140^, ^141^ However, the cycle also influences climate through its control of continental geography and through the association of supercontinent amalgamation and breakup with LIP events.^49^, ^52^, ^142^ LIP events have been correlated with a wide variety of environmental impacts and can profoundly influence global climate, both through the release of large volumes of volcanic CO_2_ to the atmosphere^143^, ^144^ and through extreme atmospheric CO_2_ drawdown brought about by the weathering of equatorial flood basalts.^145^

### Influence on global sea level

**FIGURE 5:**
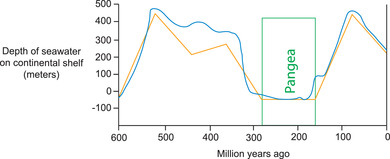
Comparison of the effect of the supercontinent cycle on sea level (straight‐segmented line), calculated for the Phanerozoic^61^ given the known duration of Pangea (box), with the first‐order eustasy curve (undulating line).^146^ The close correspondence between these two lines was used by Worsley *et al*.^61^ to support their case for a supercontinent cycle.^91^

The supercontinent cycle has a profound effect on global sea level as a result of its long‐term control of both the elevation of the continents and the depth of the ocean basins.^62^, ^63^, ^64^, ^66^ In fact, the close correspondence between the changes in global sea level predicted by the cycle for the Phanerozoic,^61^ which amounted to several hundred meters, and the contemporary depositional record of sea level change over the same interval^146^ was a key argument used in support of the original hypothesis (Figure 5). Supercontinents tend to correspond to intervals of very low global sea level^112^, ^147^ as a result of their epeirogenic uplift, either because continental insulation traps mantle heat beneath them, and/or because descent of the subduction girdle to the core–mantle boundary fosters mantle upwelling beneath them. Shortening of the crust as a result of the collisional orogenies of supercontinent assembly may also lower sea level by increasing oceanic area.^61^

Conversely, supercontinent breakup tends to correspond to a rapid global rise in sea level as a combined result of the thermal subsidence of the continental fragments as they disperse and cool, crustal extension as a result of rifting, and the decrease in ocean basin volume caused by the overall decrease in seafloor age and increase in the volume of mid‐ocean ridges that accompany the opening of new ocean basins floored by young oceanic lithosphere.^61^ This rise in sea level results in widespread continental flooding, but is ultimately reversed as the new ocean basins get older.

### Influence on atmospheric composition

**FIGURE 6:**
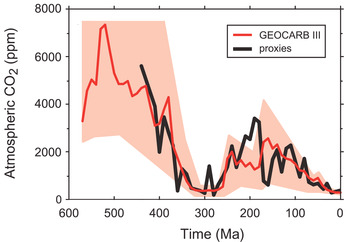
Phanerozoic proxy reconstructions and modeled predictions (Geocarb III^150^) of atmospheric CO_2_ levels for the Phanerozoic.^241^ Shaded area represents error range in modeling.

Because of its demonstrated effect on Phanerozoic global sea level, the supercontinent cycle has likely had a profound influence on the long‐term levels of CO_2_ (and other greenhouse gases) in the atmosphere (Figure 6). Atmospheric CO_2_ levels have fluctuated throughout much of Earth history in response to variations in the input of this gas from volcanic exhalations and the breakdown of carbonates and organic matter, and its removal through the chemical weathering of the continents and photosynthesis,^148^, ^149^, ^150^ the former involving its reaction with Ca and Mg silicates to form Ca and Mg carbonates following riverine transport of the weathering products to the oceans.^151^ Since the efficacy of this process depends, in part, on the land area available for chemical weathering, its effect on atmospheric CO_2_ levels, and hence climate, varies with sea level. Hydrothermal alteration of seafloor basalts likely provides an independent sink for atmospheric CO_2_,^152^, ^153^, ^154^ while the subduction of platform carbonates at continental margin arcs may provide a significant additional source.^155^

#### Supercontinent amalgamation and breakup

As a consequence of the relationship between land area and atmospheric CO_2_, supercontinents tend to coincide with climatic cooling due to atmospheric CO_2_ drawdown because they are associated with very low sea levels as a result of their thermal uplift. Adding to this cooling influence is the enhanced chemical weathering of the orogens of supercontinent assembly. Both of these processes would be amplified if true polar wander brings the supercontinent to the equator as a consequence of centrifugal forces acting on the positive dynamic topography (excess mass) created by its thermal uplift,^50^, ^54^, ^132^ since the reaction rates of chemical weathering, and hence the rate of drawdown of atmospheric CO_2_, are strongly dependent on temperature and precipitation.^76^, ^156^

**FIGURE 7:**
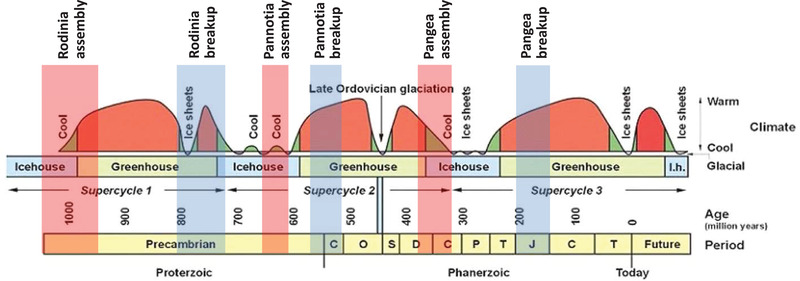
Distribution of warm (greenhouse) and cool (icehouse) global climatic conditions for the past 1 Ga^124^ compared with times of supercontinent assembly and breakup for Rodinia, Pannotia, and Pangea.

As a likely result of these processes, the amalgamation of both Pangea and Pannotia was accompanied by cold, “icehouse” climates (Figure 7) and widespread continental glaciation—respectively, the c. 335–260 Ma Gondwanan^115^, ^157^, ^158^ and the c. 640–635 Ma Marinoan^159^ and c. 580/565 Ma Gaskiers/post‐Gaskiers.^160^, ^161^, ^162^, ^163^ Conversely, continental glaciation accompanied the breakup of Rodinia (c. 717–663 Ma Sturtian^164^, ^165^, ^166^), Kenorland/Lauroscandia (c. 2.44–2.3 Ga Huronian [Gowganda]^167^, ^168^, ^169^), and perhaps even the earliest proposed supercraton Ur (c. 2.9 Ga Pongola^170^, ^171^, ^172^). This could reflect the abrupt erosional release of dissolved Ca and Mg to the oceans following the onset of rifting,^145^, ^173^ the combination of uplift and subsidence in rift settings having been long thought to provide ideal conditions for both the initiation of glaciation and the preservation of the resulting glacigenic sediments.^174^, ^175^ The role of the supercontinent cycle in continental glaciation, however, is a complex one, and while supercontinents may foster ice ages, they do not mandate them as evidenced by the apparent absence of any glaciation associated with Nuna/Columbia and its unrelated presence during the Hirnantian (c. 445 Ma)^176^, ^177^ and the Pleistocene to present day.

The Huronian glaciations accompanying Kenorland/Lauroscandia also coincide with the Great Oxidation Event (c. 2.43–2.25 Ga^178^), during which biologically produced O_2_ first started to accumulate in the atmosphere,^179^ perhaps as a result of the breakup‐related evolution of the first oxygen‐requiring cyanobacteria,^180^ or a LIP‐generated pulse of sulphate to the oceans, the reduction of which liberated oxygen.^181^ The rise in atmospheric oxygen, evident in the loss of Fe‐poor paleosols, detrital pyrite, and detrital uraninite,^182^, ^183^, ^184^ in the first appearance of redbeds,^185^ and in the loss of mass‐independent fractionation of sulfur isotopes in sedimentary rocks,^186^, ^187^ likely led to the demise of atmospheric methane, the most powerful of the greenhouse gases, thereby providing an alternative mechanism for dramatic climatic cooling.^188^, ^189^, ^190^

#### Snowball Earth

**FIGURE 8:**
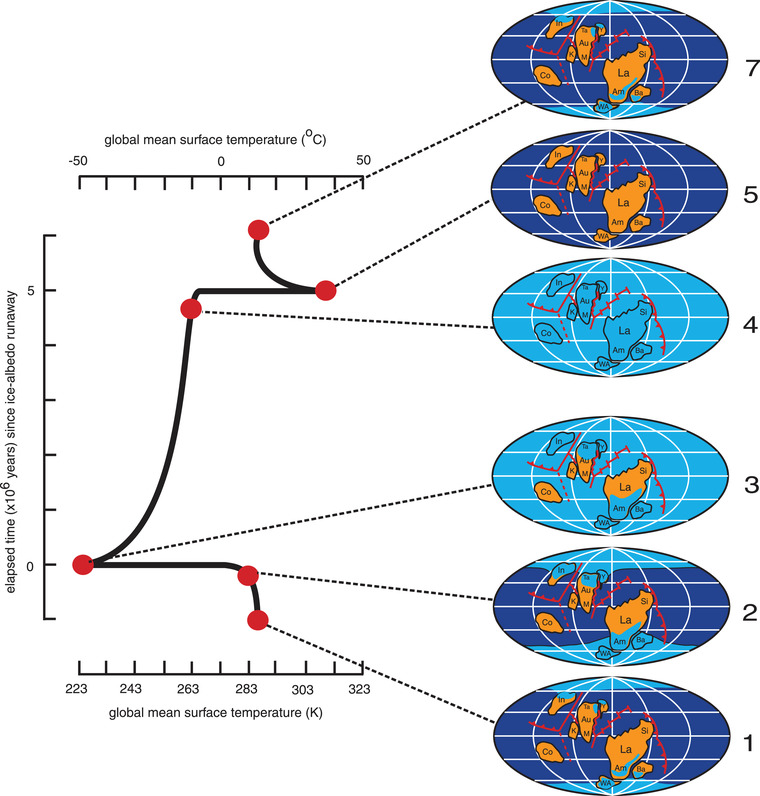
Time scale for estimated changes in global mean surface temperature, based on energy‐balance calculations, and ice extent through one complete snowball event.^192^ The global palaeogeography is for 750 Ma, some 30 m.y. before the Sturtian glaciation. Abbreviations: Am, Amazonia; Au, Australia; Ba, Baltica; Co, Congo; In, India; K, Kalahari; M, Mawson; Si, Siberia; Ta, Tarim; WA, West Africa; Y, South China

The climatic cooling that led to the continental glaciations associated with Kenorland or Lauroscandia (Huronian/Gowganda), Rodinia (Sturtian), and Pannotia (Marinoan) is thought to have been sufficiently extreme as to cause the entire planet to freeze, a unique situation known as “Snowball Earth.”^81^, ^164^, ^189^, ^190^, ^191^, ^192^ Such conditions are thought possible if ice comes to within c. 30^o^ of the equator because the albedo feedback from the planet's ice‐covered surface then becomes self‐sustaining^193^, ^194^, ^195^—one more latitudinal degree of ice cover causing albedo cooling sufficient to give one more latitudinal degree of cover (Figure 8). As a result, glacial ice spreads rapidly toward the equator, eventually leading to an ice‐covered planet with a global mean temperature estimated at c. −50°C.^192^ In the case of the Sturtian (c. 717–663 Ma^165^, ^196^) and Marinoan (c. 640–635 Ma^159^) glaciations, such Snowball Earth conditions were likely promoted by the concentration of continents between 30^o^N and 30^o^S, and the consequent high rates of chemical weathering and atmospheric CO_2_ drawdown, following the breakup of Rodinia,^145^ the final equatorial position of which^17^ may have been the result of true polar wander.^54^

Once initiated, the icehouse conditions of a Snowball Earth are thought to prevail until volcanically sourced atmospheric CO_2_, deprived by ice cover of a continental weathering (and photosynthetic) sink, rises dramatically to c. 350 present atmospheric levels.^81^, ^197^ At this threshold point, rapid greenhouse‐induced and albedo‐feedback accelerated deglaciation ensues (Figure 8), leading within c. 5 Ma, to a “hothouse” Earth with a global mean temperature of c. 40°C.^192^ With re‐establishment of the CO_2_ cycle and renewal of continental weathering (and photosynthesis), the climate rapidly returns to its initial state, setting the stage for the process to repeat. There are consequently five stages in the evolution of a Snowball Earth: (1) strong equatorial drawdown of atmospheric CO_2_ through continental weathering needed to cause the oceans to start freezing, (2) albedo‐feedback expansion of the ice cover to a latitude of c. 30^o^, whereupon it becomes self‐sustaining and the planet freezes from pole to pole, (3) shutdown of continental weathering allowing volcanically derived CO_2_ to build rapidly in the atmosphere, (4) greenhouse effect of rising atmospheric CO_2_ levels reaches a critical threshold, whereupon the ice rapidly melts and a hothouse world is established, and (5) resumption of continental weathering and restoration of the CO_2_ cycle reduces the greenhouse effect and returns climate to its initial state.

#### Supercontinent dispersal

The processes that lead to global cooling during the assembly and rifting of supercontinents are reversed following supercontinent breakup as the dispersing continental fragments cool and subside. With the ensuing rise in global sea level, the continents flood, continental weathering decreases, and atmospheric CO_2_ levels rise. As a result, continental dispersal tends to coincide with a progressive build‐up of atmospheric CO_2_ and accompanying global warming. In addition, the release of CH_4_ (or the CO_2_ produced by its oxidation) as gas hydrates break down with rising temperatures would provide this breakup‐related global warming with a strong positive feedback.^198^, ^199^ Not surprisingly, therefore, supercontinent breakup tends to coincide with climatic warming, as evidenced by the “greenhouse” climates of the Mesozoic, early Paleozoic, and much of the Tonian,^124^, ^200^, ^201^ following the breakup of Pangea, Pannotia, and Rodinia, respectively (Figure 7). The introduction of large amounts of CO_2_ into the oceans during supercontinent breakup and dispersal has also been linked to increased carbon burial and black shale abundance,^70^ while the increased run‐off of terrigenous nutrients in warmer climates has been coupled to oceanic anoxia.^202^, ^203^

An additional climatic influence of supercontinent breakup comes from its proposed link to stepwise increases in atmospheric oxygen, possibly as a consequence of enhanced marine productivity resulting from an increase in the erosional release to the oceans of nutrients, such as bioproductivity‐limiting phosphorus.^78^ Like CO_2_, atmospheric O_2_ levels are thought to have had a significant impact on long‐term global climate,^204^ even though oxygen is not a greenhouse gas. This is because rising O_2_ levels result in an increase in atmospheric density and, hence, greater scattering of incoming solar radiation and consequent reduction in surface evaporation. As a result, precipitation decreases, humidity levels fall, and cooler temperatures ensue because less heat is trapped by water vapor, which is a strong greenhouse gas.

Increased atmospheric O_2_ levels might also be expected during periods of enhanced organic carbon burial, such as those proposed to accompany the rapid sedimentation of supercontinent breakup and dispersal.^78^, ^117^ Conversely, decreased atmospheric O_2_ levels should accompany the increased chemical weathering of supercontinent amalgamation and breakup because the chemical reactions involved are largely oxidative.^204^

### Influence on mantle plumes and LIPs

**FIGURE 9:**
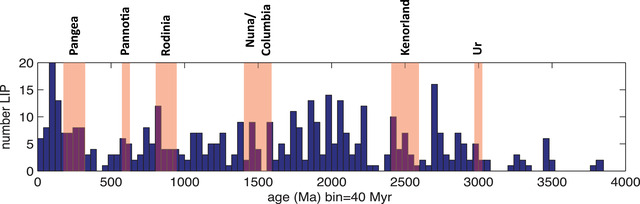
Distribution of large igneous provinces (LIPs) throughout Earth history^137^ compared with tenure of supercontinents/supercratons Pangea (c. 325–200 Ma), Pannotia (c. 620–580 Ma), Rodinia (c. 950–800 Ma), Nuna/Columbia (1.6–1.4 Ga), Kenorland (c. 2.7–2.5 Ga), and Ur (c. 3 Ga)

Since supercontinent breakup requires continents to rift, the supercontinent cycle has long been linked to mafic dike swarms and LIPs,^4^, ^49^, ^61^, ^205^, ^206^ and through their emplacement, to the activity of mantle plumes.^52^, ^58^, ^60^, ^207^, ^208^ Uncertainty continues to exist as to whether the timing of LIP events (Figure 9) coincides with the breakup of supercontinents,^4^, ^61^, ^205^, ^209^ or their amalgamation,^49^, ^206^ or both,^137^, ^138^, ^210^ in part because the timing and number of pre‐Pangean supercontinent amalgamation and breakup events remain poorly constrained^211^ even while the dating of LIP events has become increasingly precise.^51^, ^53^, ^142^ However, while evidence has been presented that questions the relationship,^138^, ^212^, ^213^ recent time‐series analysis suggests a cyclicity in both continental and oceanic LIPs and accompanying plume activity that is both comparable to that of the supercontinent cycle and corresponds closely to periods of supercontinent rifting and breakup.^60^ This is consistent with the idea that supercontinent amalgamation works to trigger mantle plumes at the core–mantle boundary;^50^, ^128^ a proposition that finds support in the correlation between the reconstructed positions of Mesozoic LIPs and the margins of the African (Tuzo) LLSVP, which has been identified at the core–mantle boundary on the basis of seismic tomography, and which is centered over the former position of Pangea.^214^, ^215^, ^216^

#### LIPs and climate

Mantle plumes can, in and of themselves, affect climate simply by thermally uplifting the lithosphere and thereby changing global sea level and weathering‐mediated atmospheric CO_2_ levels.^217^, ^218^ The influence of LIPs on global climate, however, stems from the voluminous volcanic activity with which they are associated, the effect of which can cause both climatic warming and cooling. The immediate effect of this volcanism is one of brief regional or global cooling as a result of the dispersal and absorption of solar radiation by fine volcanic ash and H_2_SO_4_ aerosols vented to the stratosphere during explosive eruptions.^219^ However, the most dramatic climatic effect of LIPs is one of long‐term global warming due to the increased magmatic venting of greenhouse gases, such as CO_2_ and CH_4_.^144^ The introduction of such gases to the atmosphere during supercontinent rifting and breakup would act to boost those generated by the decrease in continental weathering associated with breakup‐related sea level rise, further enhancing global warming. Depending on the rock‐type, contact metamorphism associated with LIP magmatism can also release huge volumes of greenhouse gases to the atmosphere.^220^ In fact, these may play a leading role in global warming, given that the dominant LIP magma is relatively gas‐poor tholeiitic basalt.

**FIGURE 10:**
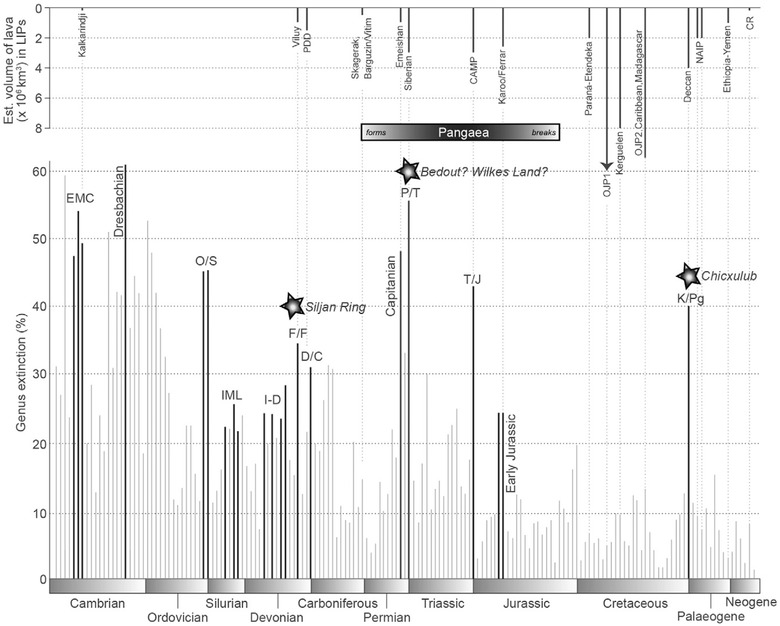
Age and estimated volume of Phanerozoic large igneous provinces (LIPs) compared to genus extinction magnitude showing correlation between mass extinction events (peaks) and LIP emplacement, particularly during tenure of Pangea.^88^ Large igneous provinces: PDD, Pripyat‐Dnieper‐Donets; CAMP, Central Atlantic Magmatic Province; OJP 1/OJP 2, Ontong Java Plateau phases 1 and 2; NAIP, North Atlantic Igneous Province; CR, Columbia River Basalt Group. Extinction events: EMC, Early to Middle Cambrian; IME, Ireviken, Mulde and Lau Events; I‐D, intra‐Devonian events; F/F, Frasnian/Famennian; D/C, Devonian/Carboniferous; P/T, Permian/Triassic; T/J, Triassic/Jurassic; K/ Pg, Cretaceous/Paleogene. Stars identify bolide impacts.

In addition to their climatic impact, LIP magmatism and contact metamorphism liberate large volumes of toxic gases, such as SO_2_ and F, so it is not surprising that Phanerozoic LIP flood volcanism has long been correlated with mass extinctions (Figure 10).^88^, ^144^, ^221^, ^222^ A strong correlation exists, for example, between the Yakutsk‐Vilyui,^223^, ^224^ Emeishan,^225^ Siberian Traps,^226^, ^227^ CAMP,^228^ Karoo‐Ferrar,^229^ and Deccan Traps^230^ LIP events and mass extinctions in the Late Devonian (Frasnian‐Famennian), Middle Permian (Capitanian), end‐Permian, end‐Triassic, Early Jurassic (Toarcian), and end‐Cretaceous, respectively.^231^ A temporal link also exists between the final pulses of the Central Iapetus Magmatic Province (CIMP)^143^ and the extinction of the Ediacaran fauna immediately prior to the Cambrian explosion.^232^

However, the long‐term warming influence of major LIP events may be followed, or interrupted, by abrupt cooling. It has been argued, for example, that the equatorial continental paleogeography of the Cryogenian, which would have favored cool global climates as a result of climate‐enhanced chemical weathering and organic carbon burial,^233^ may have been driven into runaway global glaciation of the Sturtian Snowball Earth by the weathering of extensive LIP continental flood basalts erupted throughout the break‐up of Rodinia, such as those associated with the Gunbarrel (c. 780 Ma), Mundine Well (c. 755 Ma), and Franklin (c. 723 Ma) provinces.^145^, ^234^, ^235^ Donnadieu *et al*.^145^ further suggest that this may also have been the case for the Marinoan Snowball Earth.

### Other factors

According to Jellinek *et al*.,^84^ an additional influence of the supercontinent cycle on global climate may lie in its control on the degree to which warm subcontinental mantle is globally mixed, since the impact of volcanism and weathering on Earth's long‐term carbon cycle is modulated by lateral ocean‐continent variations in mantle temperature. Their calculations suggest that supercontinents girdled by subduction zones foster lateral ocean‐continent mantle temperature variations because mixing of insulated subcontinental mantle is inhibited. As a result, outgassing of CO_2_ from mid‐ocean ridges is reduced, giving rise to cold climates and icehouse/hothouse climate variability like that associated with Rodinia. Conversely, long‐lived ice‐free climates, like that associated with Nuna/Columbia, are features of thorough mantle thermal mixing.

The supercontinent cycle can also influence climate solely as a result of the changes it makes in the distribution of continents and oceans. By applying a climate system model to the breakup of Pangea, for example, Tabor *et al*.^236^ have shown that opening of an ocean basin such as the Atlantic fosters humidification of the tropics, large‐scale reorganization of tropical circulation, and both regional and global changes in temperature. Weaker tropical easterlies and reduced upwelling warm the equatorial ocean, while increased moisture and cloud formation in the tropics cool both land and sea.

Finally, as pointed out by Foley and Driscoll,^237^ plate tectonics, as governed by the supercontinent cycle, is itself influenced by climate. Cool climates, which the cycle maintains through its plate tectonic control of the long‐term carbon cycle, act to enhance stresses within the lithosphere and promote its hydration and weakening that, in turn, enable plate tectonics to take place. Hence, the supercontinent cycle may have played a significant role in ensuring Earth maintained its status as a habitable planet.

## CONCLUSIONS

The supercontinent cycle, by which Earth history is viewed as having been punctuated by the episodic assembly and breakup of supercontinents, has, through its management of plate motion, planetary geography, sea level, and mantle circulation, profoundly influenced Earth's long‐term climatic history. By necessitating alternating episodes of supercontinent assembly, during which the continents approach one another, and breakup, during which they disperse, the cycle has governed Earth's paleogeography and, in doing so, the regional climate experienced by any given continent at any given time.^69^ By exercising control over the drawdown of CO_2_ and other greenhouse gases from the atmosphere through its influence on sea level and chemical weathering, and the input of these gases to the atmosphere through its influence on plate tectonics and magmatism, the cycle has mediated Earth's long‐term global record of alternating warm (greenhouse) and cold (icehouse) climates. A strong coupling also appears to exist between supercontinents and mantle dynamics that would link the cycle to mantle plumes and LIPs, and, consequently, the climatic effects of their volcanic emissions, which have been associated with mass extinctions, oceanic anoxia, and catastrophic changes to the surface environment. The proposed tendency for true polar wander to center supercontinents on the equator as a result of centrifugal forces acting on their excess mass may also set the stage for extreme global cooling (Snowball Earth) through the enhanced drawdown of atmospheric CO_2_ caused by the equatorial weathering of breakup‐related LIP basalts. It is, therefore, likely that the supercontinent cycle has, over the course of Earth history, played a dominant role in governing the climate of individual continents, the planet's long‐term warming and cooling trends, and its occasional climatic extremes, while, at the same time, maintaining surface conditions sufficiently hospitable to ensure the continuity of life.

## COMPETING INTERESTS

The author declares no competing interests.

### PEER REVIEW

The peer review history for this article is available at https://publons.com/publon/10.1111/nyas.14849.
